# Driving the polar spin reorientation transition of ultrathin ferromagnets with antiferromagnetic–ferromagnetic phase transition of nearby FeRh alloy film

**DOI:** 10.1038/s41598-020-71912-z

**Published:** 2020-09-10

**Authors:** P. Dróżdż, M. Ślęzak, W. Janus, M. Szpytma, H. Nayyef, A. Kozioł-Rachwał, K. Freindl, D. Wilgocka-Ślęzak, J. Korecki, T. Ślęzak

**Affiliations:** 1 Faculty of Physics and Applied Computer Science, AGH University of Science and Technology, al. Mickiewicza 30, 30-059 Kraków, Poland; 2 Jerzy Haber Institute of Catalysis and Surface Chemistry PAS, ul. Niezapominajek 8, 30-239 Kraków, Poland

**Keywords:** Magnetic properties and materials, Surfaces, interfaces and thin films

## Abstract

We show that in-plane to out-of-plane magnetization switching of a ferromagnetic layer can be driven by antiferromagnetic–ferromagnetic phase transition in a nearby FeRh system. For FeRh/Au/FeAu trilayers, the impact of the magnetic phase transition of FeRh onto the perpendicular magnetization of monoatomic FeAu superlattices is transferred across the Au spacer layer via interlayer magnetic coupling. The polar spin reorientation process of the FeAu spins driven by the magnetic phase transition in the FeRh reveals its major features; namely it is reversible and displays hysteresis.

## Introduction

Control of the spin orientation in magnetic materials is a key issue in modern spintronics^1^. Materials with a large perpendicular magnetic anisotropy (PMA) are notably important from both the application and theoretical points of view, because a large PMA provides magnetization with a high thermal stability, which is necessary for high density magnetic memories^2–7^. An example of such material are monoatomic FeAu superlattices. The layer-by-layer growth by molecular beam epitaxy (MBE) stabilizes L1_0_-type ordered phase that does not exist in Fe–Au phase diagram. The super-lattices possess intriguing magnetic properties like perpendicular magnetic anisotropy, large magnetic moments combined with a high Curie temperature^8^. In ultrathin ferromagnetic films, the direction of magnetization is routinely controlled through the spin reorientation transition (SRT) arising from the competition between various contributions to the effective magnetic anisotropy, including the shape, surface, magnetocrystalline and magnetoelastic anisotropies^9–19^. The magnetization direction can also be controlled by magnetic coupling with another magnetic film as described in the literature^20,21^. Recently, we showed that the in-plane SRT process of ultrathin Co film magnetization can be induced by the proximity of the epitaxial FeRh layer, as a result of the evolution of direct exchange coupling between the FeRh and Co spin systems accompanying the temperature-driven AFM ⇔ FM magneto-structural phase transition in the FeRh layer^22^.

In this paper we show an effective way to control the PMA of an ultrathin magnetic layer via indirect exchange coupling to the FeRh layer mediated through a thin Au spacer. For the PMA system we used Fe–Au monoatomic superlattices that are known to exhibit a square magnetic hysteresis loop and large Fe magnetic moment^8,23,24^. In our experiment, structural matching of the FeRh(001) and Au(001) surfaces allowed fully epitaxial FeRh/Au/FeAu trilayers to be grown on an MgO substrate. At low temperature, when the FeRh film is in the AFM state (denoted here as FeRh_AFM_), the interlayer magnetic coupling (IMC) between FeRh and the superlattices is weak and the FeAu stack exhibits an out-of-plane magnetization due to its intrinsic PMA. However, for the FM state of FeRh (FeRh_FM_) that can be induced by heating across the AFM ⇔ FM phase transition the FeAu and FeRh_FM_ layers are ferromagnetically coupled with in-plane magnetizations. The polar SRT process of the FeAu spins displays the major features of a magnetic phase transition in the FeRh; namely, it is reversible and hysteretic^22,25^. The phenomenon reported here goes beyond the heat-assisted magnetic recording (HAMR) concept consisting of temperature modulation of the coercivity^26,27^. In HAMR technology such thermally induced modulation of coercive field allows to write the information bit by external magnetic field generated by read–write head of the HDD. A single example of utilization of FM ⇔ AFM phase transition in FeRh in classical HAMR technology concerns FeRh/FePt system^28,29^. In our experiment we obtained a switching of the magnetization direction of FeAu that is driven purely by temperature change and without the external magnetic field. The temperature of each memory bits can be changed locally by laser heating or an electrical current flow^30^.

## Results and discussion

### Growth and structural characterization

**Figure 1:**
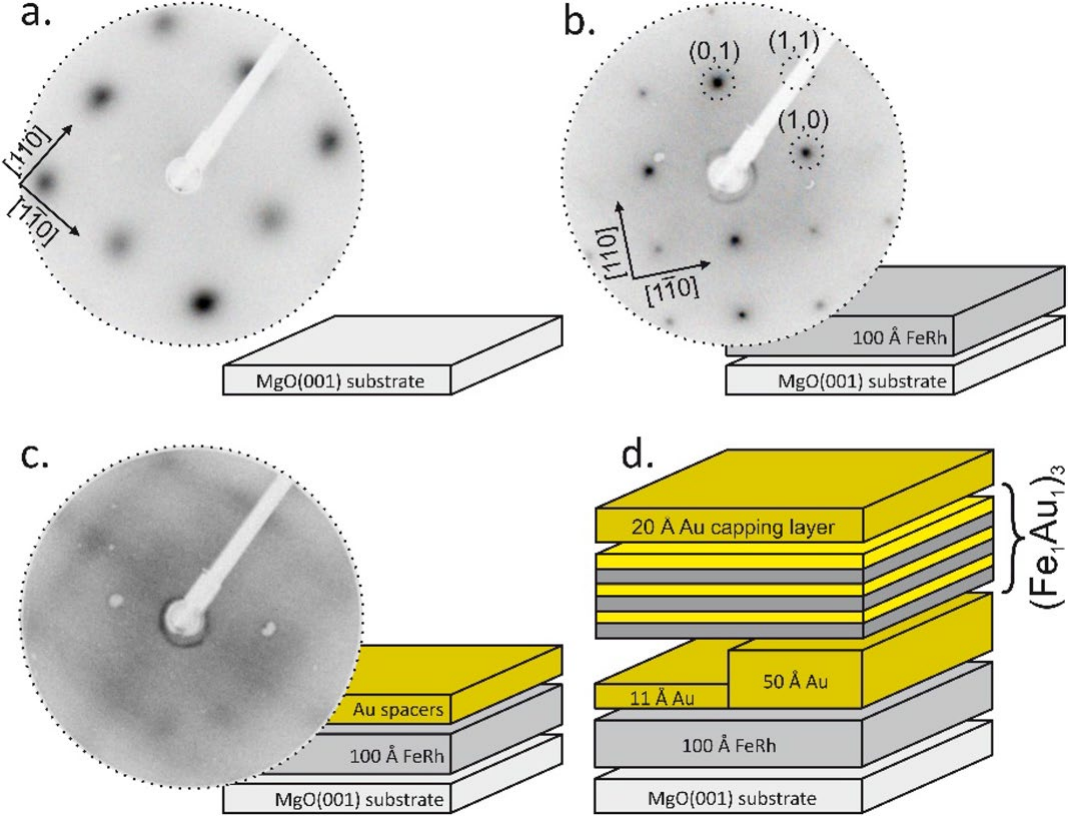
Low energy electron diffraction patterns collected for an 80-eV electron beam for (**a**) the MgO(001) substrate, (**b**) the FeRh layer and (**c**) the Au spacer. The sample architecture is schematically shown for each preparation step together with the corresponding LEED patterns. The final layer structure of the sample is shown in (**d**).

The preparation process (described in details in the “Methods” section) leads to the creation of two sample areas, FeRh/Au(11 Å)/FeAu and FeRh/Au(50 Å)/FeAu, differing only in Au spacer thickness as shown schematically in Fig. 1d. We chose the above Au thicknesses due to the following reason. Our systematic studies of the IMC in FeRh/Au/FeAu (see Supplemental Material 3) show that while for the spacer thickness of 11 Å a strong ferromagnetic interlayer magnetic coupling can be observed between FeRh and FeAu, for the Au thickness of 50 Å the IMC is negligible. Thus with a use of these two spacer thicknesses we were able to compare the magnetic properties of coupled and non-coupled layers. The sequence of the FeAu monolayers, which was repeated three times, was further denoted as the FeAu stack. The sample growth was monitored by low energy electron diffraction (LEED) at each preparation step. The diffraction patterns for the MgO(001) substrate, the FeRh layer and the Au spacer are shown in Fig. 1a–c respectively. The quality of the diffraction pattern observed for the MgO(001) substrate was low owing to the electric charging effect. The FeRh pattern is characterized by sharp diffraction spots and a low background, indicating the high structural quality of the FeRh system. Comparison of the LEED patterns of the MgO(001) substrate and the FeRh alloy indicates a 45° in-plane rotation of the MgO(001) and B2 FeRh(001) surface unit cells. Similar rotational relations were found between the fcc Au(001) and FeRh(001) surfaces. It has to be noted that despite of slight (4%) misfit between the in plane lattice parameters of FeRh(001) (a_FeRh_ = 3.00 Å) and fcc-Au(001) (a_Au_ = 2.88 Å) the epitaxial growth is preserved^25^. The diffraction pattern of the Au(001) spacer surface was weak, although traces of a typical Au(001) 5 × 1 reconstruction can be seen^31^. Finally, the similarity of the LEED patterns for the both discussed Au(001) spacers (see Supplemental Material 1), as well as for the FeAu stack grown on the two sample halves indicates homogenous structural properties of our FeAu system. The details of structural and magnetic properties of monoatomic FeAu superlattices grown on 5 × 1 reconstructed Au(001) can be found in^23,24^.

### Magnetic properties

**Figure 2:**
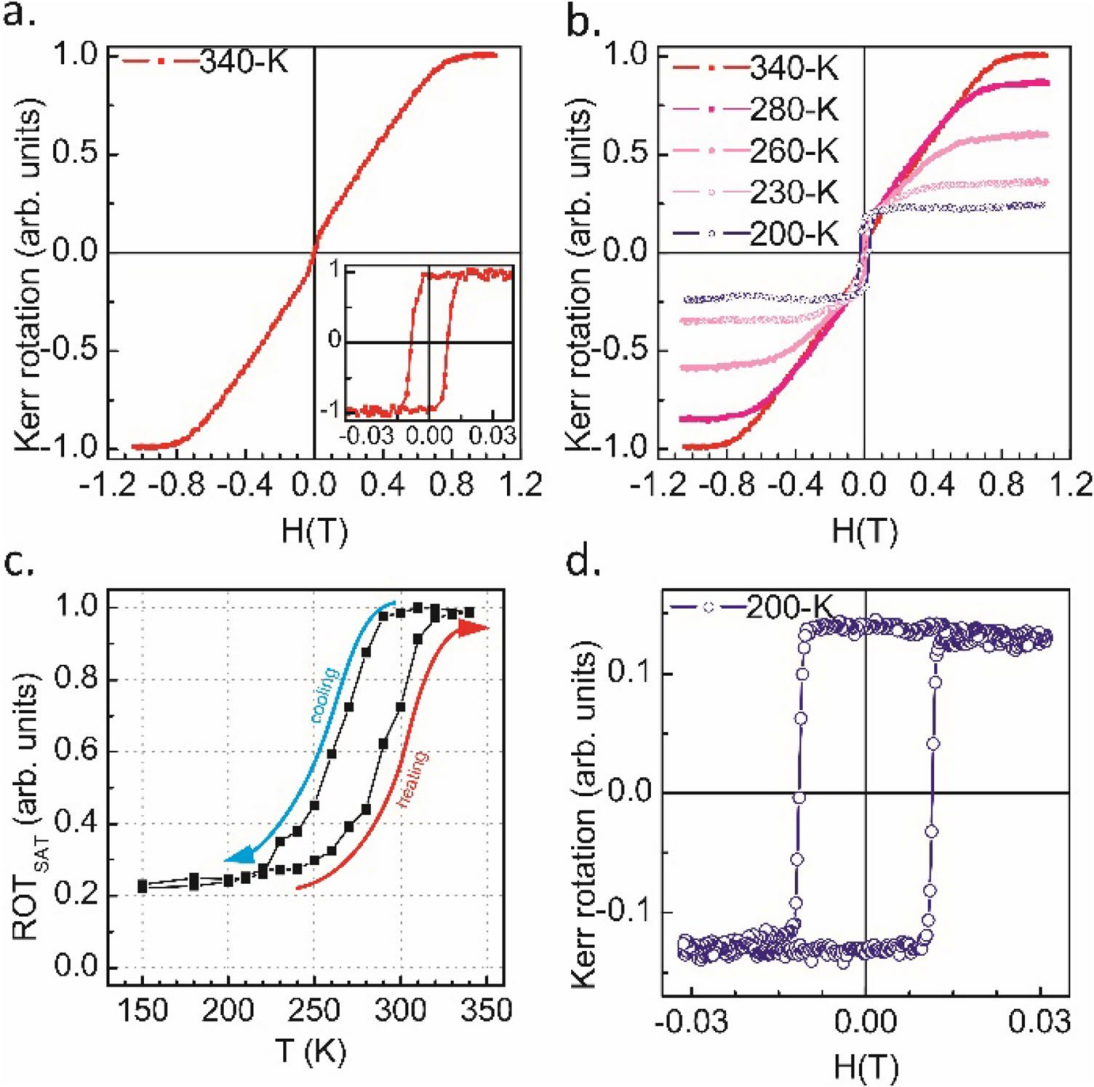
The PMOKE exemplary magnetic hysteresis loops measured for FeRh/(11 Å) Au/FeAu. (**a**) Loops measured at 340 K for polar and longitudinal (inset) geometries. (**b**) The temperature evolution of the PMOKE magnetic hysteresis loops collected during the cooling process across the FM ⇒ AFM transition. (**c**) The temperature dependence of PMOKE Kerr rotation at saturation (ROT_SAT_). Blue and red arrows indicate the cooling and heating branches respectively. (**d**) The magnetic hysteresis loop collected at 200 K in a narrower range of the external magnetic field.

The magnetic properties were studied ex situ with the polar (PMOKE) and longitudinal (LMOKE) magneto-optic Kerr^32^ effects in the temperature range between 150 and 340 K. The PMOKE and LMOKE hysteresis loops were collected during the heating and cooling processes. The exemplary PMOKE magnetic hysteresis loops measured for FeRh/(11 Å) Au/FeAu are shown in Fig. 2. The loops are normalized to the Kerr rotation at saturation determined for the loop measured at 340 K where the saturation Kerr signal has maximum value. The PMOKE loop collected at 340 K (shown in Fig. 2a) is characterized by zero remanence and a high saturation field, which in combination with a rectangular loop measured at the same temperature in the longitudinal geometry (see inset in Fig. 2a), indicates an in-plane easy magnetization direction. Figure 2b shows the temperature evolution of the PMOKE magnetic hysteresis loops collected during cooling from 340 to 200 K. The Kerr rotation at saturation (denoted here as ROT_SAT_) decreases with decreasing temperature, as shown in Fig. 2c. ROT_SAT_(T), normalized to a saturation value at 340 K, reveals the presence of the FM ⇔ AFM transition and is characterized by a typical thermal hysteresis. Remarkably, along with the FM ⇔ AFM transition manifested by a gradual decrease of the ROT_SAT_ signal with decreasing temperature, a progressive increase in the PMOKE remanence signal can be observed in the measured PMOKE loops (see Fig. 2b). Consequently, a hysteresis loop measured at 200 K in a narrower range of the external magnetic field (low-field loop), shown in Fig. 2d, is fully rectangular and corresponds to the magnetization reversal of the FeAu stack, which is in agreement with our previous observations^23^. Note that the Kerr rotation at saturation extracted from the low-field hysteresis loop measured for 200 K (see Fig. 2d) is lower than the corresponding ROT_SAT_ value determined from the loops shown in Fig. 2b. Such buildup of the saturation Kerr signal at temperatures below the FM ⇔ AFM phase transition that can be seen in Fig. 2c originates from a residual FM FeRh phase located near the FeRh/MgO interface^33^, which contributes to the ROT_SAT_ signal when the external magnetic field is high enough. A detailed discussion of the existence and origin of a residual magnetic phase in gold-capped FeRh thin films deposited on MgO is reported in our separate contribution^34^.

**Figure 3:**
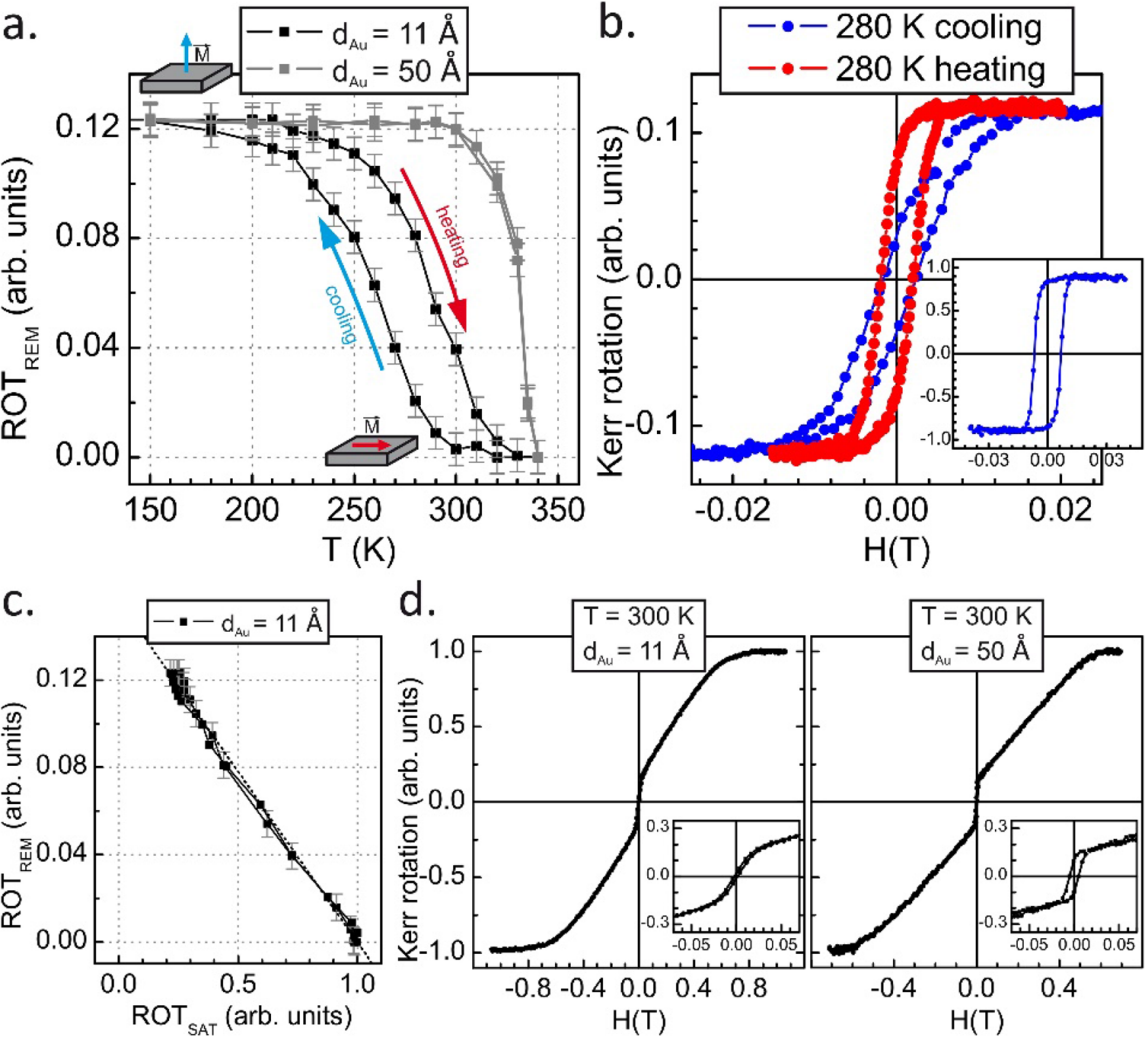
(**a**) Temperature dependence of the Kerr rotation at remanence for FeRh/(11 Å) Au/FeAu and FeRh/(50 Å) Au/FeAu. Arrows indicate heating and cooling branches (red and blue respectively). (**b**) The PMOKE loops collected for FeRh/(11 Å)Au/FeAu trilayers at 280 K during cooling (blue curve) and heating (red curve) process. The inset shows the LMOKE loop collected at 280 K during cooling process. (**c**) Dependence of the Kerr rotation at remanence as a function of the Kerr rotation at saturation with a linear fit marked by the dotted line. (**d**) The magnetic hysteresis loops collected for polar geometry at 300 K for FeRh/(11 Å) Au/FeAu and FeRh/(50 Å) Au/FeAu are shown. Insets show the zoomed central sections of these loops.

Figure 3a shows the evolution of the polar Kerr rotation at remanence (ROT_REM_) during the temperature cycle 150 K ⇔ 340 K. It is clear that at low temperatures when the FeRh alloy is in its AFM phase, the orientation of the FeRh/Au/FeAu trilayers magnetization (dominated by the contribution of the FeAu stack) is along the surface normal, while at elevated temperatures, for FeRh in the ferromagnetic state, the whole FeRh/Au/FeAu system is in-plane magnetized. It has to be noted that above analysis of the PMOKE data based on the thermal evolution of the ROT_SAT_ and ROT_REM_ parameters allows to separate MOKE contributions corresponding to the FeAu and FeRh systems. Whereas the ROT_SAT_ predominantly senses the AFM ⇔ FM phase transition in the FeRh layer, the ROT_REM_ is related only to the perpendicular magnetization component of the FeAu stacks as the PMOKE loops of Au/FeRh/MgO system do not display remanence in the 220–340 K temperature range and are characterized by high values of saturation magnetic field (~ 0.5 T) indicating that FeRh system possess intrinsically strong in-plane magnetic anisotropy in agreement with literature data^34–36^.

The straightforward interpretation of the polar SRT process of the FeAu stack involves interlayer magnetic coupling between the FeRh and FeAu spin systems mediated across the Au spacer. The IMC is negligible when the FeRh layer is in its AFM state and the FeAu stack displays out-of-plane magnetization originating from its intrinsic perpendicular magnetic anisotropy^23^. When the in-plane magnetized FM phase nucleates with increasing temperature, IMC forces the rotation of the FeAu stack magnetization to the film plane. The progressive decrease of the perpendicular magnetization component is connected to the gradual increase of the FM FeRh phase along with the transition from the AFM to FM state. The thermal evolution of the ROT_REM_ signal, which is a measure of the magnetization component perpendicular to the film plane, displays a hysteresis corresponding to the hysteresis of the FM ⇔ AFM transition in the FeRh film. Accordingly, the magnetization states of the FeAu with different spin orientations can be stabilized near room temperature depending on the thermal history of the sample. This can be directly seen from comparison of PMOKE loops collected for the FeRh/(11 Å)Au/FeAu trilayers at 280 K during cooling (blue curve) and heating (red curve) process. The hysteresis curves are clearly characterized by a significantly different values of ROT_REM_ (0.08 and 0.02 for heating and cooling, respectively) as shown in Fig. 3b. Moreover, the LMOKE loop collected at 280 K during cooling process (see inset in Fig. 3b) confirms in-plane magnetization direction of the FeRh/Au/FeAu trilayers. The slight lowering of the Kerr rotation at remanence well corresponds with the non-zero remanence in the PMOKE loop (blue loop in Fig. 3b).

Although it is not clear whether intermediate values of ROT_REM_ reflect a canted magnetization state or coexistence of magnetic domains with in-plane and out-of-plane spin orientation, an increase in ROT_REM_ is equivalent to a significant (four times) enhancement of the perpendicular magnetization component. In Fig. 3c, the dependence of the ROT_REM_ signal is plotted as a function of ROT_SAT_ for FeRh/(11 Å)Au/FeAu trilayer. The ROT_REM_(ROT_SAT_) plot was obtained from a combination of the thermal dependences of the ROT_SAT_ and ROT_REM_ parameters shown in Figs. 2c and 3a, respectively. The lack of hysteresis in ROT_REM_(ROT_SAT_) dependence indicates that the SRT of the FeAu is predominantly driven by the AFM ⇔ FM phase transition, and other magnetic phenomena, such as the temperature dependence of the magnetic anisotropy of the FeAu stack, can be neglected. These conclusions are drawn from the fact that a given fraction of the FeRh ferromagnetic phase is found across the AFM ⇔ FM phase transition at the different temperatures, T_cool_ and T_heat_, but the amount of the perpendicular magnetization component corresponding to T_cool_ and T_heat_ is identical in the ROT_REM_ (ROT_SAT_) plot. Finally, the linearity of the ROT_REM_ (ROT_SAT_) dependence indicates that the FeAu perpendicular magnetization component is proportional to the volume of the FM FeRh fraction. That in turn suggests that the interlayer magnetic coupling between the FeRh_FM_ and FeAu apparently depends linearly on the fraction of the FM phase. The nature of the effective IMC between FeAu and FeRh systems is complex and involves oscillatory RKKY-like contribution and dipolar magnetic coupling originating from stray fields generated by FeRh system. The detailed analysis of effective IMC can be found in Supplemental material 3.

The above picture of SRT in the FeAu stack suggests that in the case of the FeRh/Au/FeAu trilayer with a much thicker Au spacer, the magnetic properties of the Fe–Au superlattices should be weakly dependent on the AFM ⇔ FM phase transition in the FeRh film. PMOKE hysteresis loops measured at 300 K (on cooling from high temperature) for the FeRh/(11 Å)Au/FeAu and FeRh/(50 Å)Au/FeAu trilayers are shown in Fig. 3d. It can be seen from the insets, showing the zoomed central sections of the PMOKE loops, that the Kerr rotation at remanence is almost zero in the case of the thin Au spacer (d_Au_ = 11 Å) and becomes significant for the trilayers with the thick Au interlayer (d_Au_ = 50 Å), confirming the above interpretation that associates the SRT of the FeAu stack with IMC to the FeRh layer. Moreover, the thermal evolution of ROT_REM_ for the FeRh/(50 Å)Au/FeAu trilayers (grey curve shown in Fig. 3a) should be discussed. The ROT_REM_ value is constant over the large temperature range 80–320 K (low field loops are always rectangular in that temperature range) and drops to zero at 340 K. We believe that such behavior reveals the out-of-plane to in-plane spin reorientation transition (SRT) of FeAu stack grown on 50 Å thick Au(001) spacer on temperature increase. The exemplary PMOKE loops and discussion of its shape evolution as a function of temperature can be found in the Supplemental Material 2. We note a lack of temperature hysteresis of the transition from out-of-plane to in-plane magnetization direction of the FeAu system. Such SRT, is probably induced by a thermal weakening of perpendicular magnetic anisotropy of FeAu system (magnetic surface anisotropy), however it can be also driven by a combined effect of weaker perpendicular magnetic anisotropy and residual magnetic coupling with in-plane magnetized FeRh system.

## Conclusions

We demonstrated that the magnetization orientation of a ferromagnetic film can be switched between the in-plane and out-of-plane directions by the AFM ⇔ FM phase transition in a nearby FeRh system. In the case of the FeRh/Au/FeAu trilayers, the impact of the FeRh magnetic state on the magnetization of the FeAu superlattices is transferred via interlayer magnetic coupling across the Au spacer layer. The reported phenomenon provides a mechanism for writing information purely by the temperature change. The dependence of IMC on the spacer thickness is an additional factor that enabled control of the magnetization state in the studied system. Our observations can be important from the point of view of heat-assisted magnetic recording.

## Methods

### Sample preparation

The 100-Å-thick FeRh layer was grown on a polished MgO(001) single crystal by elemental co-deposition at 670 K. Fe was evaporated from a resistively heated BeO crucible, while the evaporation of Rh was achieved by electron bombardment. The pressure during deposition was around 5 × 10^–10^ mbar. The nominal Rh atomic concentration established by adjustment of the Fe and Rh evaporation rates (both in the range of Å/min) was approximately 54%. The base FeRh layer was post-annealed at 1,000 K for 1 h to promote the formation of the desired B2 structure. Next, Au spacers with two different thicknesses of 11 Å and 50 Å were deposited at room temperature on the substrate halves. The monoatomic superlattices were grown on the top of the Au spacer by alternate deposition of Fe(001) and Au(001) monolayers (ML). The thicknesses of the Fe and Au ML were 1.43 Å and 2.04 Å respectively and the sequence of the Fe–Au monolayers was repeated three times. For ex-situ measurements, the sample was capped with a 20-Å Au protective layer. The thicknesses of the films were determined with using a quartz microbalance cross calibrated with X-ray reflectivity data. The precision of the thickness determination can be estimated to 5% of monolayer.

### Structural properties: LEED

The low-energy electron diffraction (LEED) technique was used to investigate the atomic structure of the growing layers at every preparation steps. More detail about growth and structural properties of (FeAu) stacks on Au(001) spacers can be found in Supplemental Material 1.

### Magnetic properties: MOKE

The magnetic properties were studied ex situ with the polar (PMOKE) and longitudinal (LMOKE) magneto-optic Kerr effects. The combination of the both PMOKE and LMOKE techniques provides an access to the both in-plane and out-of-plane magnetization component of samples and its thermal evolution. We used a standard lock-in detection setup consisting of an s-polarized laser light source (*λ* = 635 nm) and a photo-elastic modulator with a modulation frequency of 50 kHz. The second harmonic (2f.) signal measured by the detector is proportional to the Kerr rotation, and was taken as a measure of the magnetization. The MOKE method probes the entire FeRh/Au/FeAu system as shown in^34^. During measurements the sample was attached to the copper holder inside the flow cryostat placed between magnetic poles. The temperatures were controlled by a resistance heater and flow rate of nitrogen.

## Supplementary information

Supplementary Information 1.

Supplementary Information 2.

Supplementary Information 3.
