# Supplementary material for: Driving the polar spin reorientation transition of ultrathin ferromagnets with antiferromagnetic–ferromagnetic phase transition of nearby FeRh alloy film
Source: Sci Rep. 2020 Sep 10;10:14901. doi: 10.1038/s41598-020-71912-z (PMC7484764; doi:10.1038/s41598-020-71912-z)
Supplement: Supplementary file 1 — Supplementary Information 1. [file 41598_2020_71912_MOESM1_ESM.docx]

**Driving the polar spin reorientation transition of ultrathin ferromagnets with antiferromagnetic-ferromagnetic phase transition of nearby FeRh alloy film**

P. Dróżdż^1^^[[1]](#footnote-1)^, M. Ślęzak ^1^, W. Janus ^1^, M. Szpytma ^1^, H. Nayyef ^1^, A. Kozioł-Rachwał ^1^, K. Freindl^2^, D. Wilgocka‑Ślęzak^2^, J. Korecki ^1,2^, T. Ślęzak  ^1^

^1^AGH University of Science and Technology, Faculty of Physics and Applied Computer Science, al. Mickiewicza 30, 30-059 Kraków, Poland

^2^Jerzy Haber Institute of Catalysis and Surface Chemistry PAS, ul. Niezapominajek 8, 30-239 Kraków, Poland

**Growth and structural properties of (FeAu) stacks on Au(001) spacers**

The LEED patterns of Au(001) spacer are very similar for the both discussed Au thicknesses as shown in upper panel of figure S1. Moreover, the LEED patterns acquired after deposition of third (top) Fe monolayer of FeAu stack (see bottom panel of figure S1) are also very similar for the FeAu grown on the discussed Au spacers. This indicates that epitaxial growth of FeAu stacks is very similar on thinner and thick Au spacer.


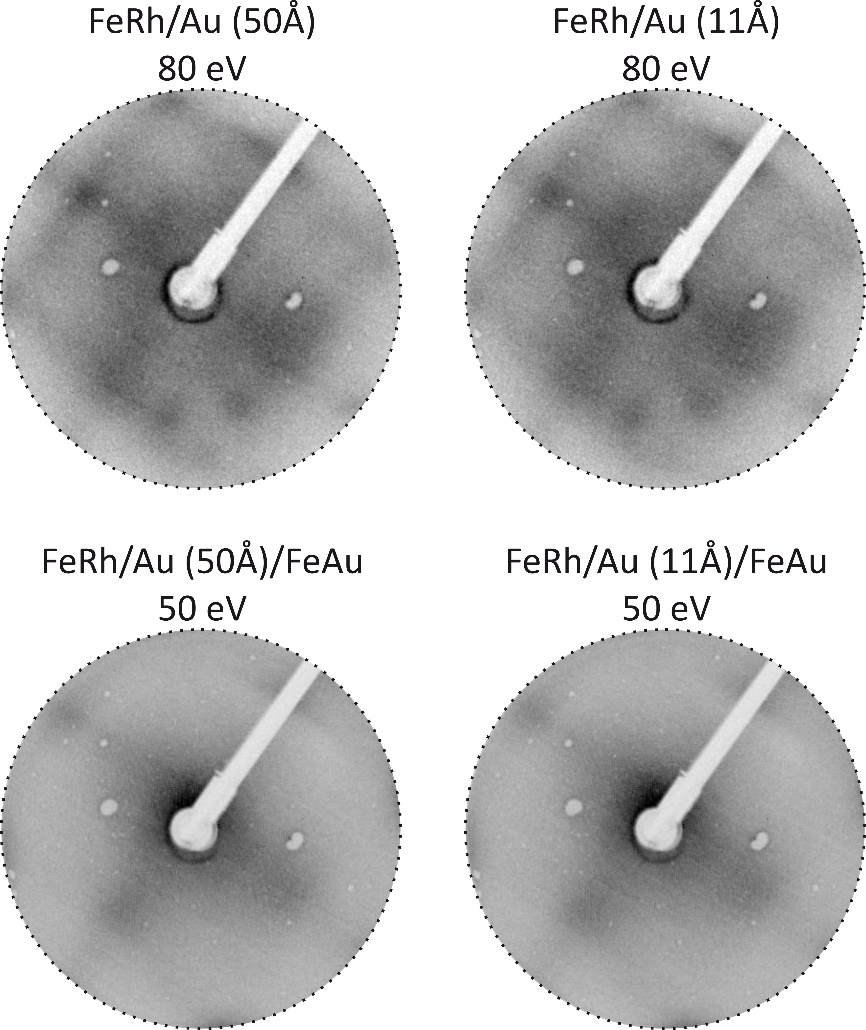


Fig. S1 The LEED patterns acquired for the Au(001) spacers surfaces (upper panel) for the energy of incident electrons E=80eV. In the bottom panel the LEED patterns of FeAu monoatomic superlattices grown on Au(001) spacers acquired for the energy of incident electrons E=50eV are shown.

1. email: piotr.drozdz@fis.agh.edu.pl [↑](#footnote-ref-1)
