# Supplementary material for: Driving the polar spin reorientation transition of ultrathin ferromagnets with antiferromagnetic–ferromagnetic phase transition of nearby FeRh alloy film
Source: Sci Rep. 2020 Sep 10;10:14901. doi: 10.1038/s41598-020-71912-z (PMC7484764; doi:10.1038/s41598-020-71912-z)
Supplement: Supplementary file 2 — Supplementary Information 2. [file 41598_2020_71912_MOESM2_ESM.docx]

**Driving the polar spin reorientation transition of ultrathin ferromagnets with antiferromagnetic-ferromagnetic phase transition of nearby FeRh alloy film**

P. Dróżdż^1^^[[1]](#footnote-1)^, M. Ślęzak ^1^, W. Janus ^1^, M. Szpytma ^1^, H. Nayyef ^1^, A. Kozioł-Rachwał ^1^, K. Freindl^2^, D. Wilgocka‑Ślęzak^2^, J. Korecki ^1,2^, T. Ślęzak  ^1^

^1^AGH University of Science and Technology, Faculty of Physics and Applied Computer Science, al. Mickiewicza 30, 30-059 Kraków, Poland

^2^Jerzy Haber Institute of Catalysis and Surface Chemistry PAS, ul. Niezapominajek 8, 30-239 Kraków, Poland

**The temperature driven spin reorientation transition of FeRh/(50 Å)Au/FeAu trilayers**

In Fig. S2 the low field PMOKE loops (that reveal magnetization reversal of FeAu only) measured for FeRh/(50 Å)Au/FeAu system are shown for selected temperatures during heating process. It has to be noted that identical shape of the loops was found during cooling at corresponding temperatures. The curves are rectangular in 300 – 330 K range and become typical for a ferromagnet with in-plane magnetization at 340 K. Such behavior reveals the out-of-plane to in-plane spin reorientation transition (SRT) of FeAu stack grown on 50 Å thick Au(001) spacer on temperature increase. Such SRT, is most probably induced by a thermal weakening of perpendicular magnetic anisotropy of FeAu system (magnetic surface anisotropy), however it can be also driven by a combined effect of weaker perpendicular magnetic anisotropy and residual magnetic coupling with in-plane magnetized FeRh system.


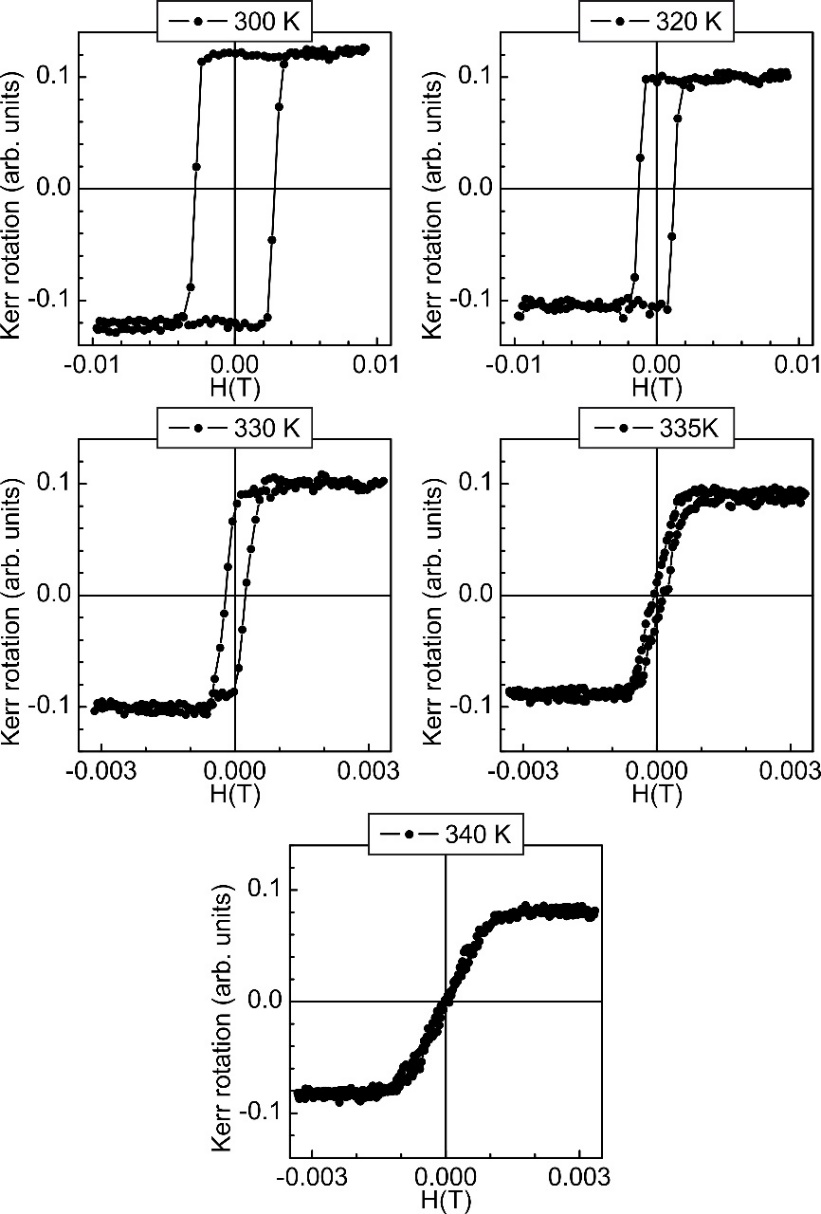


Fig. S2 Exemplary low field PMOKE loops collected for FeRh/(50 Å)Au/FeAu system during heating process.

1. email: piotr.drozdz@fis.agh.edu.pl [↑](#footnote-ref-1)
