# Supplementary material for: Driving the polar spin reorientation transition of ultrathin ferromagnets with antiferromagnetic–ferromagnetic phase transition of nearby FeRh alloy film
Source: Sci Rep. 2020 Sep 10;10:14901. doi: 10.1038/s41598-020-71912-z (PMC7484764; doi:10.1038/s41598-020-71912-z)
Supplement: Supplementary file 3 — Supplementary Information 3. [file 41598_2020_71912_MOESM3_ESM.docx]

**Driving the polar spin reorientation transition of ultrathin ferromagnets with antiferromagnetic-ferromagnetic phase transition of nearby FeRh alloy film**

P. Dróżdż^1^^[[1]](#footnote-1)^, M. Ślęzak ^1^, W. Janus ^1^, M. Szpytma ^1^, H. Nayyef ^1^, A. Kozioł-Rachwał ^1^, K. Freindl^2^, D. Wilgocka‑Ślęzak^2^, J. Korecki ^1,2^, T. Ślęzak  ^1^

^1^AGH University of Science and Technology, Faculty of Physics and Applied Computer Science, al. Mickiewicza 30, 30-059 Kraków, Poland

^2^Jerzy Haber Institute of Catalysis and Surface Chemistry PAS, ul. Niezapominajek 8, 30-239 Kraków, Poland

**The mechanism of magnetic interlayer coupling in FeRh/Au/FeAu system**

In order to verify the dependence of perpendicular magnetization of the FeAu superlattice on the spacer thickness we have prepared a dedicated MgO(001)/FeRh/Au/FeAu sample with a special Au spacer, including a wedge part, 0 to 30 Å, and a flat, 50 Å thick part. The Au spacer thickness dependences of normalized ROT_REM_ determined from PMOKE loops are shown for selected temperatures in Fig. S3a.


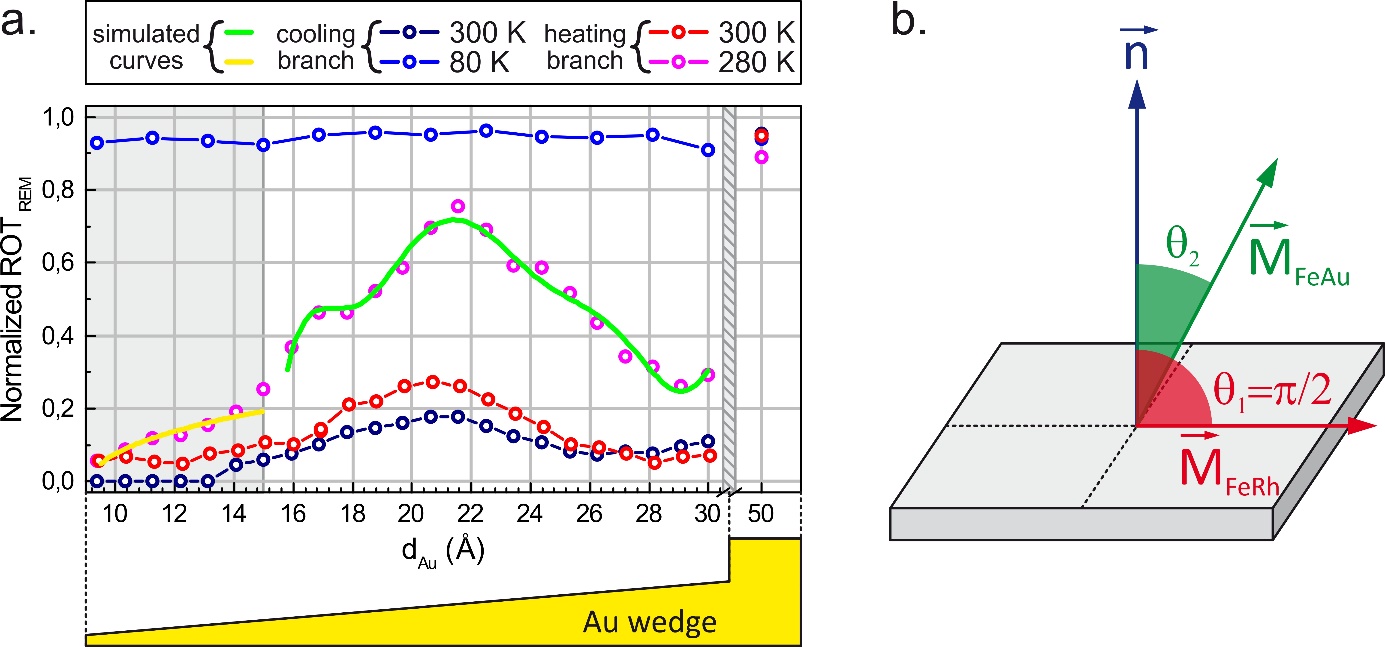


Fig. S3 a) Au spacer thickness dependences of normalized ROT_REM_ for selected temperatures for cooling and heating branches, b) the angles $\theta_{1}$and $\theta_{2}$with respect to the sample normal defining orientation of FeRh and FeAu magnetizations are shown.

At low temperature (80 K), corresponding to the AFM state of the FeRh alloy, the ROT_REM_(d_Au_) dependence is flat with average value above 0.9 in whole Au thicknesses range which corresponds to nearly rectangular PMOKE loops. At this temperature range the interlayer magnetic coupling (IMC) is negligible owing to inconsiderable amount of the FM phase in the FeRh system. Consequently, high remanence values arise from intrinsic perpendicular magnetic anisotropy of the FeAu superlattices. It has to be noted that a small amount of the residual ferromagnetic phase of FeRh that is present at low temperatures in the nominally AFM state is located at the FeRh/MgO interface as shown in^1^ and is decoupled from a FeAu system. The absence of interlayer magnetic coupling between residual FM phase of FeRh arise from its spatial separation from Au/FeAu bilayer by FeRh layer in the AFM phase.

At a mixed AFM/FM state of the FeRh, that is reached via heating from 80 K to 280 K the magnetic coupling between ferromagnetic FeRh and FeAu stacks is complex and its origin can be interpreted in the following way. For the ultrathin Au spacer thickness 0 < d_Au_ < 15 Å (shaded area in the Fig. S3a) a strong collinear coupling is most probably related to the pinholes existing in the Au spacer that originate from the structural imperfections at the FeRh/Au and Au/FeAu interfaces. Strong ferromagnetic interlayer magnetic coupling at pinhole sites can dominate effective coupling^2^ and leads to the rotation of FeAu magnetization towards the film plane that is manifested by lowered ROT_REM_ signal. For Au spacers thicker than 15 Å the ROT_REM_ signal vs d_Au_ displays clearly a non-monotonous behavior, that as shown below, is related to the long and short periods oscillations of the interlayer exchange coupling (IEC). In order to derive a theoretical dependence of the ROT_REM_ parameter on the Au spacer thickness d_Au_ we assumed the following expression for the free energy per unit volume of FeRh/Au/FeAu system:

$E\left( \theta_{1},\theta_{2} \right)=K_{1}{cos}^{2}\theta_{1}-K_{2}{cos}^{2}\theta_{2}+J_{eff}cos(\theta_{1}-\theta_{2})$,

where $\theta_{1,2}$ are the angles between the sample normal and magnetizations of FeRh and FeAu layers as shown in the Fig. S3b. The $K_{1}$ and $K_{2}$ are the effective magnetic anisotropy constants for FeRh and FeAu layers, respectively. The last term in the $E\left( \theta_{1},\theta_{2} \right)$ expression corresponds to the effective magnetic coupling energy between FeRh and FeAu magnetizations with strength $J_{eff}(d_{Au})$. In order to determine FeRh anisotropy energy density $K_{1}$ the anisotropy field $H_{A}^{FeRh}=8000 Oe$ was derived from PMOKE loop (Fig. 2b). Next, we assume the bulk value of the saturation magnetization ($M_{s}$) which for our stoichiometry is equal to $800\frac{emu}{{cm}^{3}}$ ^3^. The assumption about bulk-like properties of 10 nm thick FeRh film is justified by its bulk‑like hyperfine magnetic field values for both FM and AFM phases as shown in^1^. As mentioned above the strong in-plane magnetic anisotropy of FeRh is independent on the temperature and thickness of Au spacer. Such conclusion can be drawn taking into account a very high values of anisotropy field $H_{A}^{FeRh}=8000 Oe$ of FeRh film at 280 K (for both cooling and heating branches) and constant value of saturation magnetization across AFM⇔FM transition that can be judged from the thermal evolution of magnetic hyperfine field of FM FeRh phase derived from our Mössbauer spectra. As seen from Fig. S4b that $B_{HF}$ is almost identical across AMF⇔FM transition on its cooling and heating branches. The following equation was used to calculate anisotropy energy density for both FeRh and FeAu films:

$K=\frac{M_{s}H_{A}}{2}$.

Hence the anisotropy energy density $K_{1}=3.2\cdot{10}^{6}\frac{erg}{{cm}^{3}}$. To obtain the Fe/Au anisotropy energy density $K_{2}$ we analysed SQUID data measured for the single (Fe_1_Au_1_)_3_ stack sandwiched between Au(001) films. The SQUID magnetic hysteresis loops collected for external magnetic field applied in the film plane is shown in the Fig. S4a. The saturation magnetization $M_{S}=480\frac{emu}{{cm}^{3}}$ and anisotropy field $H_{A}^{FeAu}=3400 Oe$ were determined from the hard-axis SQUID loops. Hence the $K_{2}=8\cdot{10}^{5}\frac{erg}{{cm}^{3}}$. The comparison of $K_{1}$ and $K_{2}$ values indicates much stronger in-plane magnetic anisotropy energy of the FeRh system than out-of-plane magnetic anisotropy of FeAu stack. Thus, in the following it can be assumed that free energy of the system depends only on the $\theta_{2}$ angle ($\theta_{1}=\frac{\pi}{2}$). Such assumption is also in agreement with the literature data showing that although anisotropy field of FeRh system varies across the AMF⇔FM transition the strong in-plane effective magnetic anisotropy is preserved^4^.


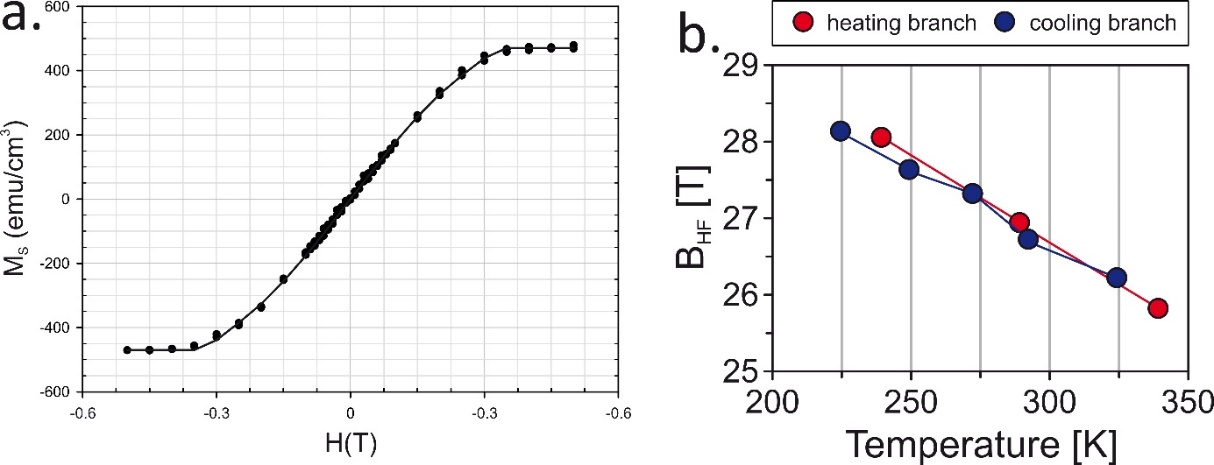


Fig. S4 a) SQUID magnetic hysteresis loop collected for external magnetic field applied along the surface of the sample. b) The temperature dependence of magnetic hyperfine fields $B_{HF}$ of FM component derived from Mössbauer spectra as a function of temperature.

In the ultrathin Au spacer thickness regime (d_Au_ < 15 Å) the dependence of the pinhole coupling strength on the Au spacer thickness can be expressed according to the Bobo et al.^2^ by the $J\left( d_{Au} \right)={C\left( \frac{1}{d_{Au}} \right)}^{c}$. The minimization of the free energy with respect to $\theta_{2}$ ($\frac{\partial E}{\partial\theta_{2}}=0)$ gives:

$${ROT}_{REM}\left( d_{Au}<15 Å \right)=\sqrt{1-\left( \frac{J\left( d_{Au} \right)}{2K_{2}} \right)^{2}}.$$

In Fig. S3a the experimental ROT_REM_ signal can be well reproduced by the above given theoretical model by adjusting the C, c and K_2_ values (see yellow curve corresponding to the theoretical ROT_REM_ parameter).

For thicker spacers 15 Å < d_Au_ < 30 Å the energy of effective magnetic coupling $J_{eff}(d_{Au})$, consists of the bilinear exchange coupling $J_{ex}(d_{Au})$ with two components oscillating as a function of Au thicknesses with a long $\left( T_{1} \right)$ and short $\left( T_{2} \right)$ oscillations periods superimposed on the non-oscillatory interlayer coupling of dipolar origin. Hence the effective coupling between ferromagnetic FeRh and FeAu layers is given by:

$J_{eff}\left( d_{Au} \right)={A\left( \frac{1}{d_{Au}} \right)}^{2}\cos\left( 2\pi\frac{d_{Au}}{T_{1}} \right)+B\left( \frac{1}{d_{Au}} \right)^{2}\cos\left( 2\pi\frac{d_{Au}}{T_{2}} \right)+{C\left( \frac{1}{d_{Au}} \right)}^{c}$,

where $A, B, C, c$ parameters influence the coupling strength and its decay with increasing spacer thickness. Again the minimization of the free energy over $\theta_{2}$ leads to the numerical expression for ROT_REM_ parameter:

$${ROT}_{REM}\left( d_{Au}>16 Å \right)=\sqrt{1-\left( \frac{J_{ex}\left( d_{Au} \right)+J\left( d_{Au} \right)}{2K_{2}} \right)^{2}}$$

The green line in Fig. S3a corresponds to the fit of the experimental ROT_REM_ vs d_Au_ data with its above given numerical expression of ROT_REM_. The satisfying match of the theoretical ROT_REM_ curve and measured ROT_REM_(d_Au_) dependence at 280 K could be obtained for $T_{1}=17 Å$ and $T_{2}=5.3 Å$ in a good agreement with the long (8.6 Au(001) ML) and short (2.5 Au(001) ML) oscillations of periods of IEC for Au spacer predicted by theory^5^

Finally, the effective interlayer magnetic coupling as function of the thickness of the Au spacer (see Fig. S5a) can be calculated from ROT_REM_ equation assuming$K_{2}=8\cdot{10}^{5}\frac{erg}{{cm}^{3}}$ and further decomposed into the oscillatory RKKY-like $J_{ex}$ and no oscillatory contribution $J_{dip}$^2,6^. It can be seen that strength of the oscillatory RKKY-like coupling varies between $-0.03\frac{erg}{{cm}^{3}}$and $0.04\frac{erg}{{cm}^{3}}$ while the dipolar contribution is much stronger ranging from $0.18\frac{erg}{{cm}^{3}}$ to $0.14\frac{erg}{{cm}^{3}}$ in the investigated Au spacer range. The nature of the effective magnetic coupling between FeAu and FeRh systems is complex and involves oscillatory RKKY-like contribution and dipolar magnetic coupling originating from stray fields generated by FeRh system. At the mixed AFM/FM FeRh phase transition stages (for example at 280 K on heating branch) the stray magnetic field originate from the discontinuous patches of ferromagnetic phase. However, for pure FM state of FeRh the dipolar magnetic field probably results from roughness of the FeRh film. At higher temperatures (300 K) the oscillatory coupling mechanism becomes weaker thus the oscillations of ROT_REM_ signal vs spacer thickness are blurred. Further, for 50 Å thick Au spacer the effective magnetic coupling between FeRh and FeAu systems vanishes and remanence reaches value close to unity for all discussed temperatures.


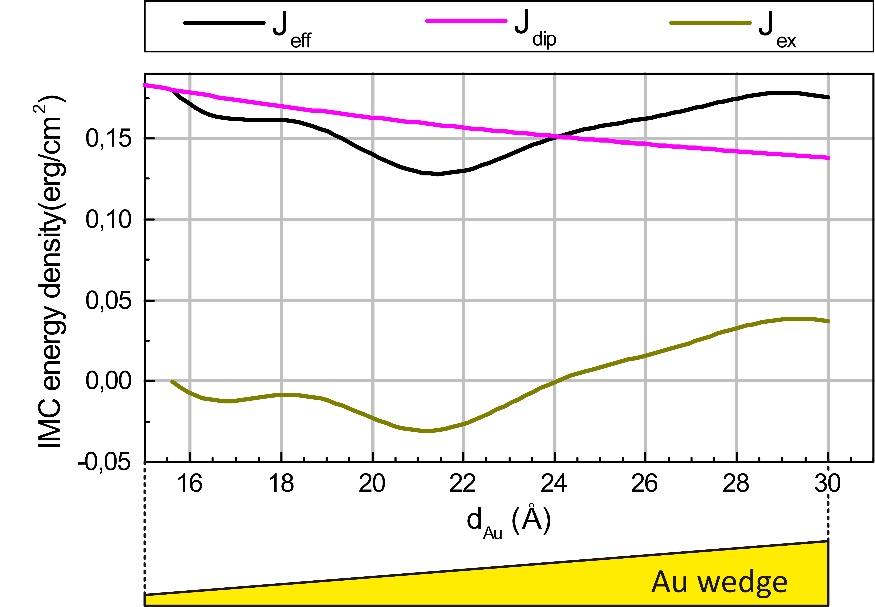


Fig. S5 The thickness dependence of the interlayer magnetic coupling energy density. The effective, dipolar and interlayer exchange coupling components were shown as black, magenta and green lines, respectively.

1. Dróżdż, P. *et al.* Perpendicular magnetic anisotropy and residual magnetic phases in gold- capped FeRh film on MgO(001). *J. Magn. Magn. Mater.* **495,** 165804 (2020).

2. Bobo, J. F. *et al.* Pinholes in antiferromagnetically coupled multilayers: Effects on hysteresis loops and relation to biquadratic exchange. *Phys. Rev. B* **60,** 4131 (1999).

3. Inoue, S., Ko, H. Y. Y. & Suzuki, T. Magnetic properties of single-crystalline FeRh alloy thin films. *IEEE Trans. Magn.* **44,** 2875–2878 (2008).

4. Wang, Y. *et al.* Spin pumping during the antiferromagnetic–ferromagnetic phase transition of iron–rhodium. *Nat. Commun.* **11,** 1–8 (2020).

5. Bruno, P. & Chappert, C. Oscillatory Coupling between Ferromagnetic Layers Separated by a Nonmagnetic Metal Spacer. *Phys. Rev. Lett.* **67,** 1602–1605 (1991).

6. Demokritov, S. O., Tsymbal, E., Grunberg, P. & Zinn, W. Magnetic-dipole mechanism for biquadratic interlayer coupling. *Phys. Rev. B* **49,** 720–723 (1994).

1. email: piotr.drozdz@fis.agh.edu.pl [↑](#footnote-ref-1)
